# Successful targeting in situ of an oncogenic nuclear antigen by hapten induced tumor associated autoantibodies (iTAA)

**DOI:** 10.1038/s41598-023-36757-2

**Published:** 2023-06-19

**Authors:** Baofa Yu, Jian Zhang, Qiang Fu, Yan Han, Jie Zhang, Feng Gao, Peng Jing, Peicheng Zhang, Guoqin Zheng

**Affiliations:** 1 TaiMei Baofa Cancer Hospital, Dongping, 271500 Shandong Province China; 2 Jinan Baofa Cancer Hospital, Jinan, 250000 Shandong Province China; 3 Shandong University, Jinan, 250000 Shandong Province China; 4 Beijing Baofa Cancer Hospital, Beijing, 100010 China; 5 Immune Oncology Systems, Inc, San Diego, CA 92102 USA; 6 South China Hospital of Shenzhen Univisity, 518055 Shenzhen, China

**Keywords:** Biological techniques, Cancer, Chemical biology, Drug discovery, Immunology, Neuroscience

## Abstract

The abscopal is a hypothesis for treating of non-irradiated tumors after localized radiation therapy. It is associated with the products of tumor-associated gene as autoantibodies (aTAAs) in reaction to the tumor-associated antigens (TAAs), with increasing of anti-MAGEA3 and an relationship between the abscopal effect and immune response. The hapten enhanced local chemotherapy (HELC) was studied to kills tumor and release tumor TAAs, then hapten modify the TAAs to neu-TAAs, to produce tumor autologous antibodies, called induced tumor-associated autoantibodies (iTAAs) that is different from natural TAAs. Since the iTAAs and complement (C) are associated with cancer therapy Immunofluorescence (IF) was applied to evaluate the expression of the iTAAs and C3, C5, C9. Traces resulted in a partial staining of the nucleus in C3’s perinuclear reaction. The iTTAs of Survivin, C-MYC, and IMP1 increased significantly in the tumor cells' intranuclear regions (P = 0.02, P = 0.00, P < 0.0001). Koc, zeta, RalA, and p53 had a similar trend in the perinuclear regions (P < 0.0001, P = 0.004, P < 0.0001, P = 0.003). Therefore, we can propose that tumor antigens inside the cancer cells’ nuclei are targeted by the iTAAs since the iTAAs binding levels are higher after HELC. The iTAA tagging oncogenic nuclear antigens may play a distinctive role in regulating tumor cell growth.

## Introduction

Since 1953, Mole mentioned a phenomenon abscopal effect in the phrase more than half a century, the concept of abscopal effect is a hypothesis for treating metastatic cancer after local radiation therapy and the mechanism of abscopale effect is still unknown^1^. However, a promising new research was observed that abscopal is associated with products of tumor-associated gene expression as autoantibodies (aTAAs) in reaction to the tumor-associated antigens (TAAs), with increasing of anti-MAGEA3 after localized radiation therapy, and an relationship between the abscopal effect and an immune response, reappearance and immune response to the system is called abscopal effect which may eventually help us understand the key to metastatic cancer and find a way of how to reduce and treat the potentially metastasis of cancer^2–5^. Since complement is associated antibodies of TAA and has come into play with a great potential as effector system for cancer immunotherapy, so in this study complement is studied as well as TAA and aTAA^5,6^.

aTAAs combined with miRN was used to examine esophageal squamous cell carcinoma (ESCC)^7^. The experiment yielded significant results which validated the diagnostic capability for a combination of aTAAs and miRNAs to predict ESCC in patients at an early stage^7,8^. A previous publication demonstrated that TAAs plus hapten could stimulate the immune system to control tumor shrinkage and keep survival time longer due to DC, CD4 and CD8 increases in tumor tissue and a higher expression of Collal, CD4, IL12aÂ, TGFb1Â, Elastin, NFKB, Cox2, CD11c, CD8 and TNFaÂ in tumor, which are induced by hapten enhanced local chemotherapy (HELC) kill tumor and produce an *neu* TAAs^4,9,10^. These newly higher expression of immune associate genes was observed thorough DC, T cells systems, B cell systems must be involved in the immunity reaction as same time as T cell in the immunity reaction induced by HELC. We believed that the hapten modify the TAAs as neu TAAs to produce tumor autologous antibodies (iTAAs) that is different from natural aTAAs^7,11^.

Research about aTAAs operating as biomarkers for disease detection predominantly relates to the occurrence and recovery of tumors^7,11–14^. There is no research published on where the aTAAs or iTAAs circulate after they are produced and how this circulation may connect with their relationship to curative treatments or abscopal. However, the iTAAs share a connection with therapeutic cancer treatments and may play into the distal abscopal effect of tumors^9,15,16^. Hence, a preliminary investigation of the iTAAs in cancer patients revealed an increase in autologous antibodies of TAA: HCC1, RalA, zeta, and p16^17^. These results point toward the rationale that the increase may be related to extend survival time with the abscopal effect^9,18^. So far, scientists do not know how to track iTAAs that result from distal tumor cells or if the iTAAs can enter tumor cells. If they are to enter the tumor cells, researchers do not know wheather the iTAAs’ ability to enter the cells is associated to the complement response which opens the tumor membrane.

We specifically selected representatives of zeta, IMP1, Koc, Survivin, c-MYC, RalA, and p53 gene as marker for research, each gene may have different function in the tumorgenesis, among them, the p53 cancer suppressor gene was a very hot gene studied in the last century, we aimed to provide evidence to prove that HELC treatment induces tumor responses in cancer patients to produce iTAA. Furthermore, we aimed to decipher if the interdependence of tumor cell membranes is an adjuvant function of complement and if the iTAAs induced in the body travel to tumor cells. If so, we intended to determine which location. Finally, we sought to determine whether the iTAAs enter tumor cells at their primary tumor of primary stage or later during metastasis. This focus was set in an effort to advance our understanding of the relationship between iTAAs and abscopal effect.

## Materials and methods

### Clinical specimens

The patients received HELC treatment at Taimei Baofa Cancer Hospital. Each had a precise clinical diagnosis, met the indications for HELC chemotherapy, signed the informed consent form, and this experiment was approved by the hospital ethics committee Taimei Baofa Cancer Hospital (TMBF 0010, 2015) for therapy and participation in the study prior to either commencing and all method for experiments were performed in accordance with relevant guidelines and regulations^4,17^.

A total of seven patients with tumors were included in the study. The cases included three non-small cell lung cancer (NSCLC) patients, two esophageal squamous cell carcinoma (ESCC) patients, one cervical squamous cell carcinoma patient, and one left parotid gland malignancy patient. Each received an HELC treatment which consists of combination and off label use with adriamycin, cytarabine, hydralazine as hapten, final concentration is 1.0 mg + 0.8 mg + 1.0 mg + 7.2 mg/ml (Total dose 5 ml), the injection needle tip in the tumor was monitored by CT^4,9,10^. Biopsy samples were collected by biopsy needle from all of the patients one weeks before and one to two weeks after their HELC treatment, all sample is very small and limited to section more than 10 slides so that sometimes IF staining cannot performed for each sample by 7 tumor antigens.

To observe the abscopal effect, the biopsy sites were the core of untreated tumor or metastased lymph node after primary tumor HELC treatment for three NSCLC, one cervical squamous cell carcinoma, and two ESCC, one left parotid gland malignancy (Table 1). A few untreated tumor samples before HELC treatment is used for control. Once biopsied, the clinical specimens were immediately preserved in formalin, embedded in paraffin, and sectioned for IF staining while some patient’s blood collected for measure the level of iTAA.

**Table 1:** Participant Baseline Characteristics.

Serial number	1	2	3	4	5	6	7
Enrolled patients ID	22,757	22,356	22,356	22,845	22,284	22,751	24,603
Sex	Male	Male	Male	Male	Male	Male	Female
Age	77	59	67	68	63	79	53
KPS	80	75	87		80	87	90
Diagnosis	Esophagus	Malignant tumor in left parotid gland	Lung cancer at left side	lung cancer at right site	lung cancer at right site	Metastasis of Esophagus	Cervical cancer
Cigarette smoking	×	**√**	**√**	×	**√**	**√**	×
Alcohol intake	√	**√**	**√**	**√**	**√**	**√**	×
Stages of disease	*Stage III*	*Stage III*	*Stage VI*	*Stage III*	*Stage III*	*Stage III*	*Stage III*
Pathology: adenocarcinoma (A) squamous carcinoma (B)	B	A	A	B	B	B	B
*Prior chemotherapy*	√	√	√	√	√	√	×
*Prior adjuvant therapy*	√	√	√	√	√	√	√
*Locally advanced*	**√**	√	√	√	√	√	√

### Antibody detection analysis

An enzyme-linked immunosorbent assay (ELISA) was used to assess the signals of 7 purified recombinant proteins in phosphate-buffered saline (PBS). The final concentrations ranged from 0.125 ug/ml to 1.0 ug/ml. The proteins were then coated in a 96-well microliter plate (100ul/well) overnight at 4 °C and incubated in a 1:200 diluted serum in antigen-coated wells (100ul/well) for 90 min at room temperature (RT). Each well's optical density (OD) value was immediately read at 405 nm on the Varioskan LUX Multimode Microplate Reader to reduce the plates' variation^19,20^.

### Reagents for staining

Complement C3 primary antibody (ab11871):Mouse monoclonal (755) to C3/C3b; secondary Antibody (ab150115):Goat Anti-Mouse IgG H&L (excitation wavelength, Ex: 652 nm, Em: 668 nm, red); Clonal antibody for complement C5 (ab219387):sheep polyclonal antibody to C5. Secondary antibody (ab150177):Donkey Anti-Sheep IgG H&L (Ex: 495 nm, Em: 519 nm, green), and Rabbit monoclonal [EPR11232-82] to C9 (ab173302); Secondary antibody (ab150078): Goat Anti-Rabbit IgG H&L (Ex: 555 nm, Em: 565 nm, red) kits were purchased from Abcam. The zeta, IMP1, Koc, Survivin, c-MYC, RalA, and p53 of TAAs were synthesized with fluorescence by Beijing Yiqiao Shenzhou Technology Co., Ltd (Em: 540 nm, green).

#### IF staining: complement

C3, C5, C9 fluorescent dyeing process: (1) Baked slices: 65℃ one hour (2) Conventional dewaxing: ① Xylene two times, 15 min each ② 100% ethanol two times, 5 min each ③ 90% ethanol 5 min ④ 70% ethanol 5 min ⑤ double-distilled water 5 min (3) EDTA antigen retrieval: boiled the EDTA antigen retrieval solution on high heat (Power:100%) in a microwave oven, inserted the slides into the antigen retrieval solution, turned on the high heat (Power:100%) from for 30 s to low heat (Power:25%) 15 min, and then cooled them in the water 20 min until room temperature (4) Blocking: 3% goat serum was blocked at 37 °C 30 min (5) Primary antibody incubation: diluted primary antibody (1:200) with 1% goat serum overnight at 4 °C (6) Rewarming: warmed at room temperature 30 min and washed with TBST 3 times, 5 min each. (7) Secondary antibody incubation: diluted the secondary antibody in PBS solution (1:1000), incubated at 37 °C 30 min, and washed the slides three times with TBST, 5 min each. (8) Nuclei staining: nuclear stained with DAPI 15 min. Washed the slides three times with TBST 5 min each. The slides were mounted with an anti-fluorescence quencher and stored at 4 °C^21^.

#### IF staining: TAA

TAA-bearing fluorescein staining : Steps (1)–(3) are the same as complement fluorescence staining. (4) Permeabilized with 0.1% Triton X 100 15 min and washed the slides with TBST 5 min. (5) Blocking: blocked with 3% BSA (3gBSA + 100 ml PBS), 37 °C 30 min. (6) Incubated with TAA-bearing fluorescein, using 1% BSA (1gBSA + 100 ml PBS) to dilute the TAA-bearing fluorescein (1:500) at 37 °C 30 min, and washed the slides three times with TBST, 5 min each time. (7) Nuclei staining: nuclear staining with DAPI 15 min. Washed the slides three times with TBST 5 min each. Mounted the slides with an anti-fluorescence quencher and stored them at 4 °C^21^.

#### IF: imaging and analysis

After staining was complete, each section was photographed at Shandong University with a multispectral panoramic tissue scanning microscope (TissueFAXS Spectra). An individual blinded to this study and had no conflict of interest performed this photography and then conducted the fluorescence imaging and data analysis. The Tissue FAXS Viewer software was used for processing, and the images were exported after adjusting the lower and upper values of the image range before and after treatment to be consistent^21^.

### Statistical analysis

The expression differences of complement factors, as well as the factors of iTAAs were analyzed in the tissues before and after treatment using GraphPad Prism v8.0.2.263.21 A paired t-test was used to determine the percentage of positive cells and immunofluorescence intensity (MFI) and P < 0.05 indicated a statistically significant difference.

## Results

### Autologous antibodies of TAAs

It is found a differences in the levels of IMP1, Koc, p62, RalA, Survivin, Zeta, NPM1, Cmyc, p53, HCC, and p16 with statistically significant before and after HELC (*P* < 0.05) (Table 2, Fig. 25), it showed the level of iTAA increase in sera after HELC therapy.

**Table 2:** Comparison of serum antibodies before and after treatment.

Antibody type	Case 1 (left lung cancer)		Case 2 (right lung cancer)		Case 3(Metastasis of Esophagus)	
	Before treatment	After treatment	Before treatment	After treatment	Before treatment	After treatment
Cmyc	0.203	0.253	0.199	0.222	0.187	0.229
IMP1	0.263	0.290	0.279	0.290	0.226	0.258
Koc	0.268	0.278	0.280	0.285	0.196	0.271
p53	0.146	0.144	0.304	0.294	0.126	0.277
RalA	0.203	0.184	0.204	0.239	0.148	0.192
Survivin	0.145	0.146	0.153	0.152	0.129	0.202
Zeta	0.193	0.184	0.165	0.176	0.140	0.164

### Positive complement cells and iTAAs’ mean immunofluorescence intensity (MFI) rates

**Figure 1:**
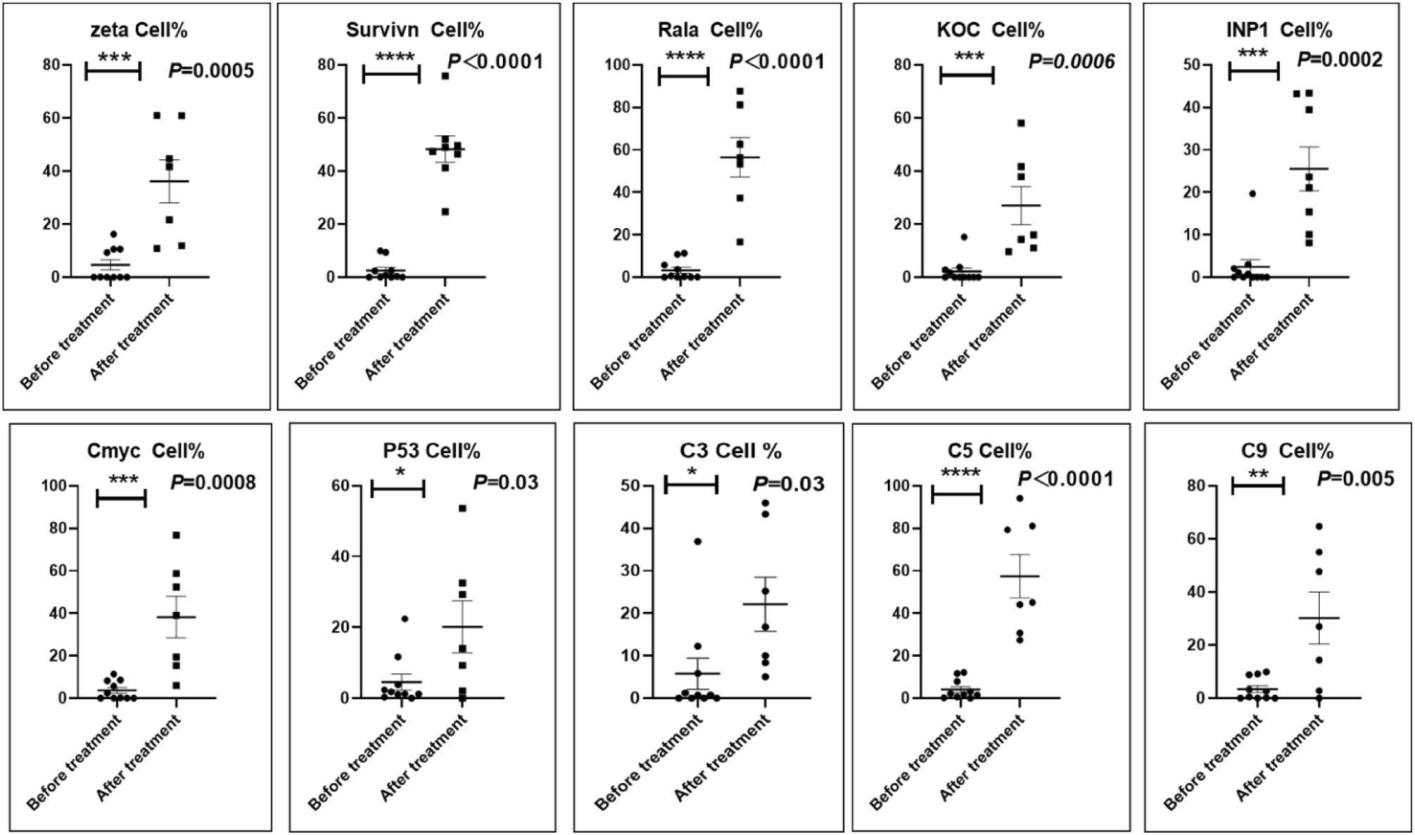
Complement positive reaction and target binding rate of tumor-related immune autoantibodies positive cells before and after treatment. *P < 0.05; **P < 0.01; ***P < 0.0001.

**Figure 2:**
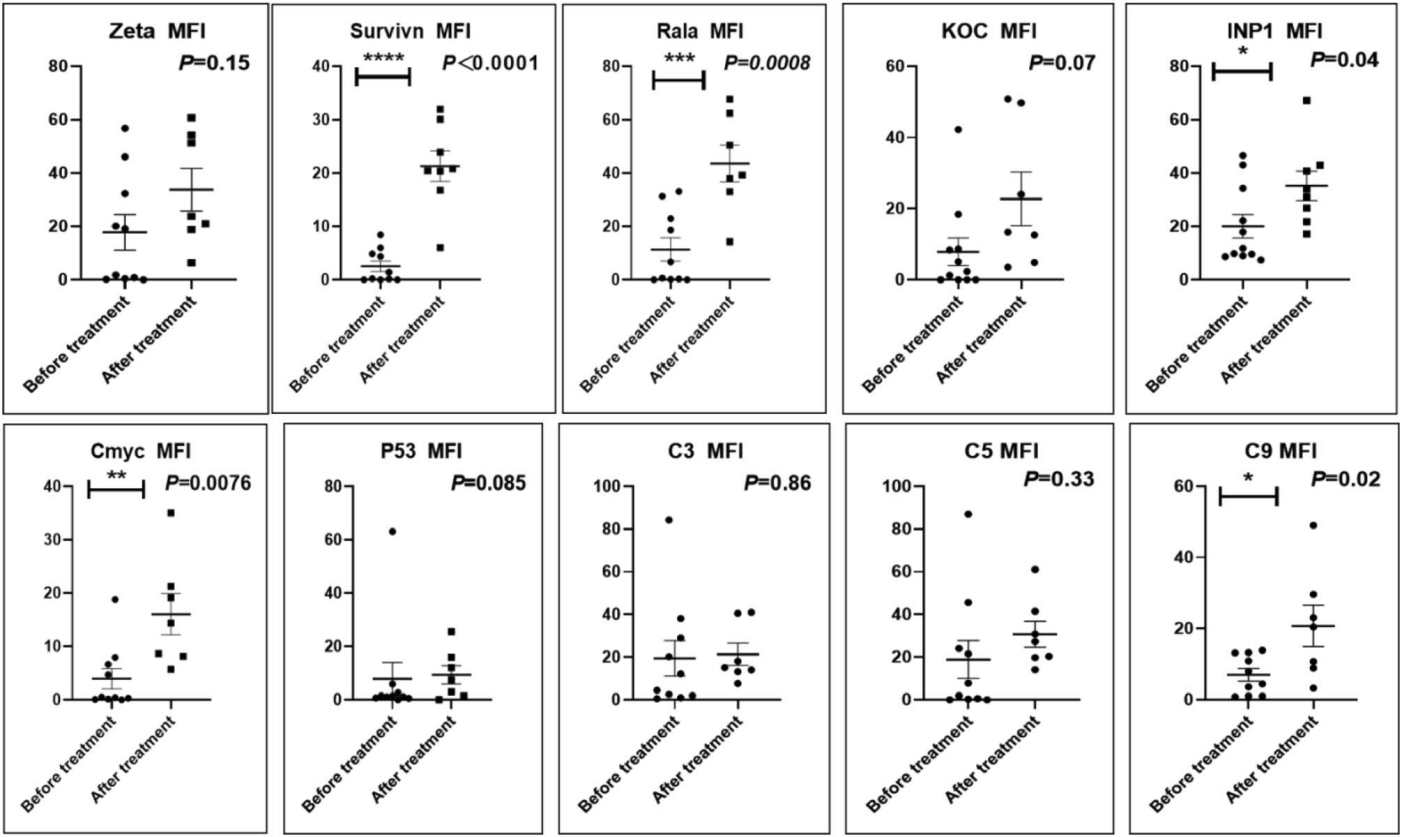
Mean immunofluorescence intensity (MFI) of complement and tumor-associated immune autoantibodies before and after treatment. *P < 0.05; **P < 0.01; ***P < 0.0001, *N* Nuclear, *C* Cytoplasm.

**Figure 3:**
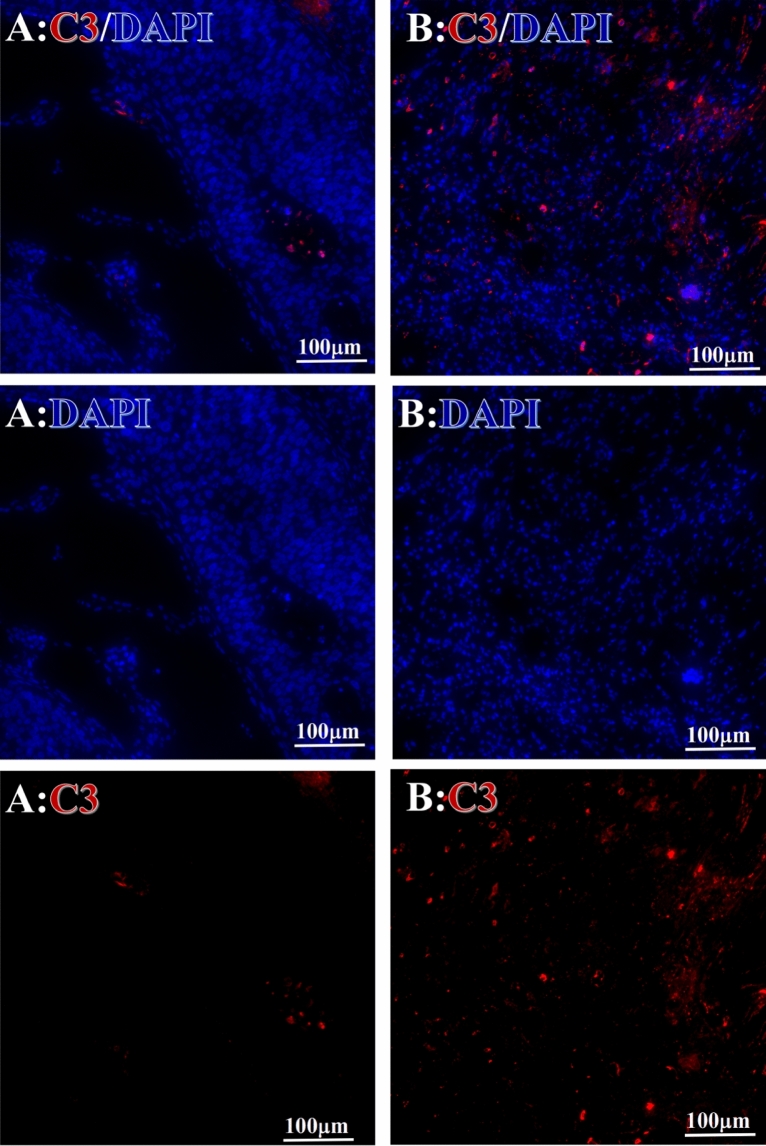
Example graph of C3 fluorescence reaction before and after HELC treatment. C3: red, DAPI: blue. A: Pathology No. WPY-6, cervical cancer, biopsy sample from primary tumor before HELC treatment; B: Pathology No. 18026, esophageal cancer (small cell carcinoma), biopsy sample from abdominal lymph node metastasis which is not treated after HELC treatment.

**Figure 4:**
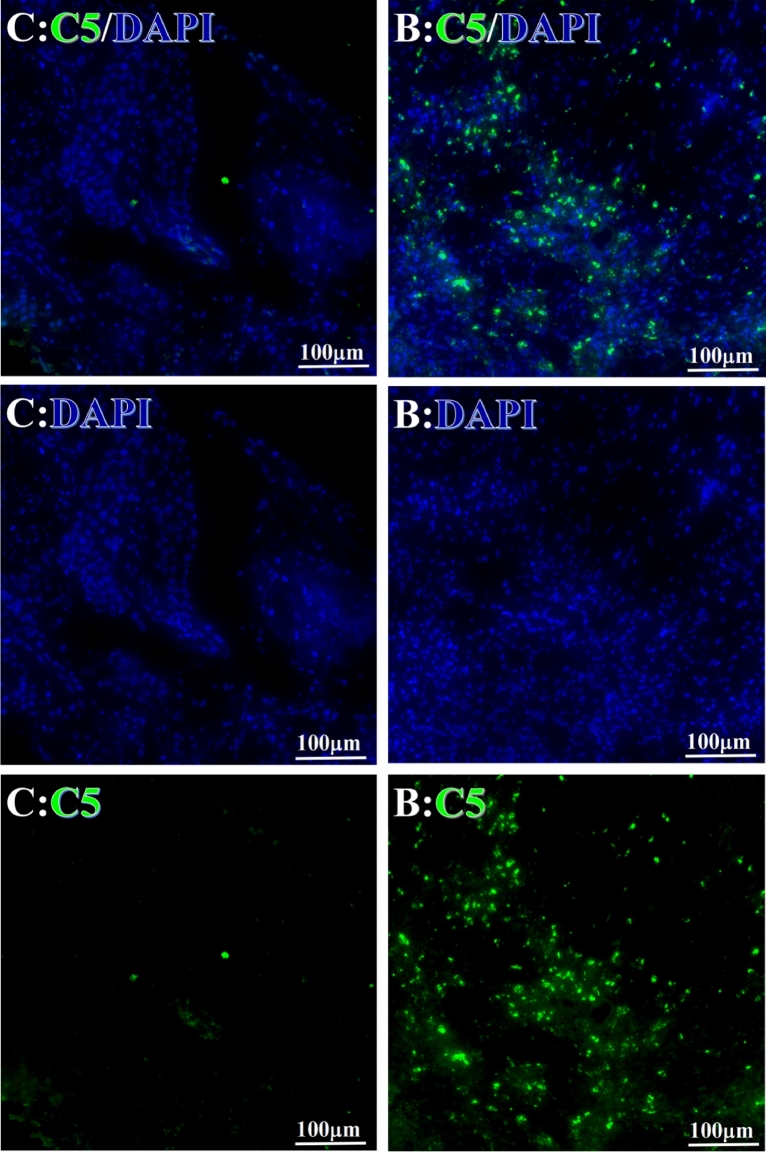
Example graph of C5 fluorescence reaction before and after primary tumor HELC treatment. C5: Green, DAPI: blue. C: Pathology No. 18050, right lung cancer, biopsy sample from the primary tumor before HELC treatment; B: Pathology No. 18026, esophageal cancer (small cell carcinoma), biopsy sample from abdominal lymph node metastasis which is not treated after primary tumor HELC treatment.

**Figure 5:**
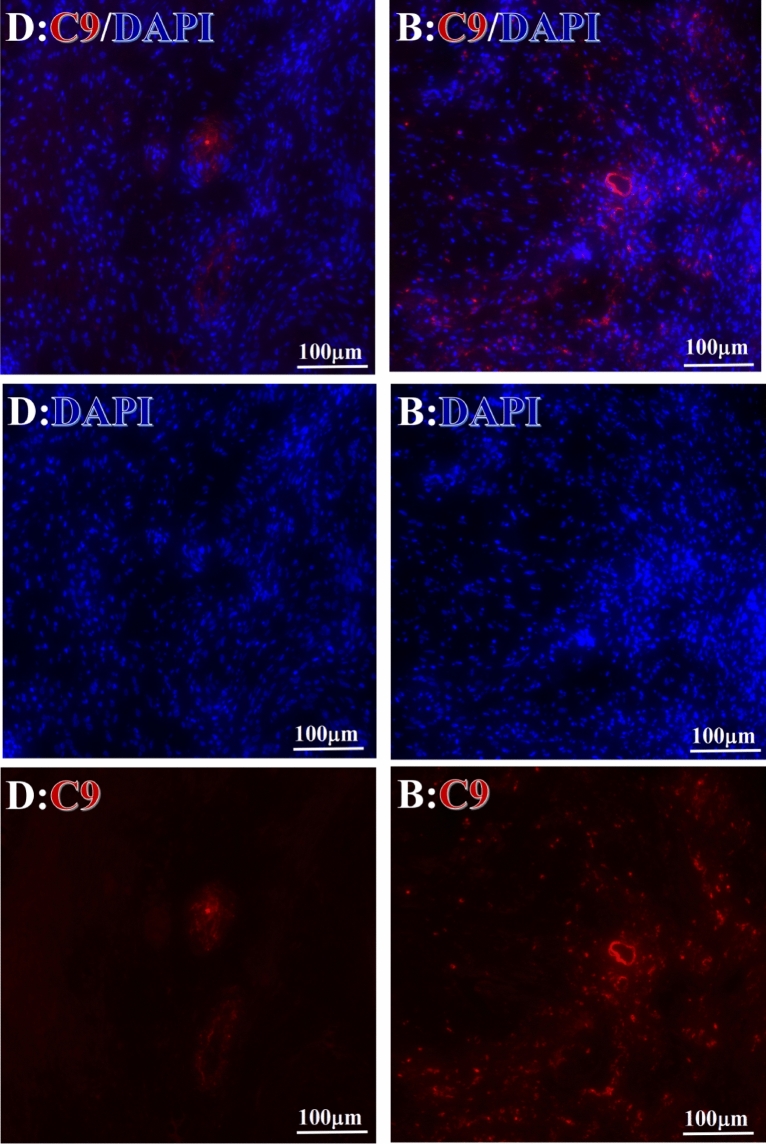
Example graph of C9 fluorescence reaction before and after primary tumor HELC treatment. C9, red, DAPI: blue. D: Pathology No. WPY-3, cervical cancer, biopsy sample from the primary tumor before HELC treatment, B: Pathology No. 18026, after treatment with sustained-release library, esophageal cancer (small cell carcinoma), biopsy sample from abdominal lymph node metastasis which is not treated after primary tumor HELC treatment.

**Figure 6:**
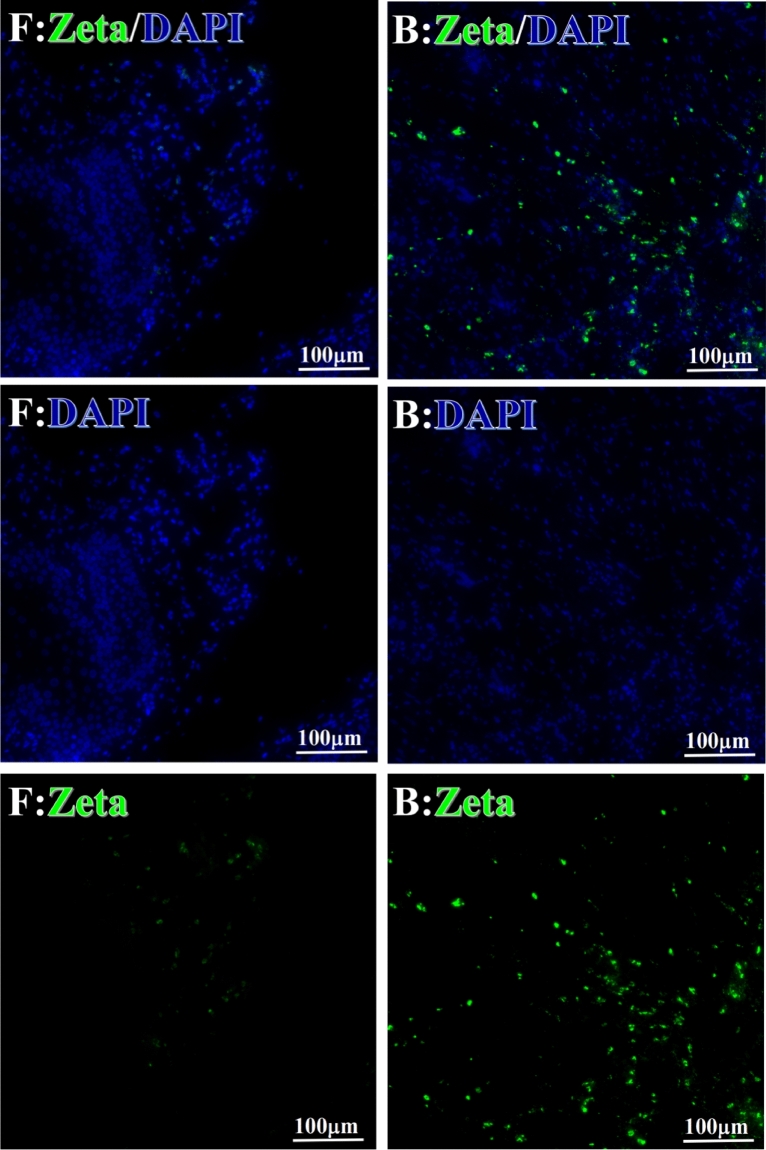
Examples iTAA of Zeta fluorescence target binding positive cells before and after primary HELC treatment. Zeta: Green, DAPI: blue. F: Pathology number 18023, esophageal squamous cell carcinoma, biopsy sample from the primary tumor before HELC treatment. B: Pathology number 18026, esophageal cancer (small cell carcinoma), biopsy sample from lymph node metastasis in the abdominal cavity which is not treated after primary tumor HELC treatment.

**Figure 7:**
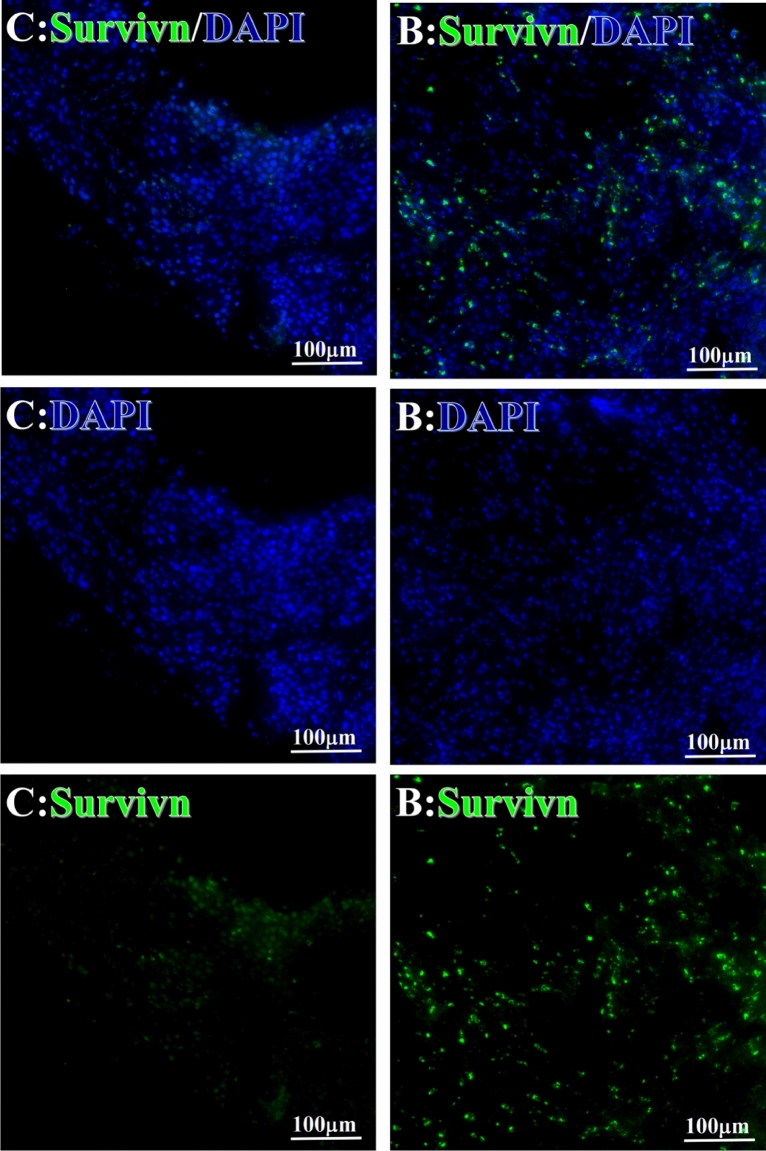
Examples iTAA of Survivn fluorescence-target binding positive cells before and after primary tumor HELC treatment. Survivn: Green, DAPI: blue. C: Pathology No. 18050, right lung cancer, biopsy sample from the primary tumor before HELC treatment; B: Pathology No. 18026, esophageal cancer (small cell carcinoma), biopsy sample from the abdominal lymph node metastasis which is not treated after the primary tumor HELC treatment.

**Figure 8:**
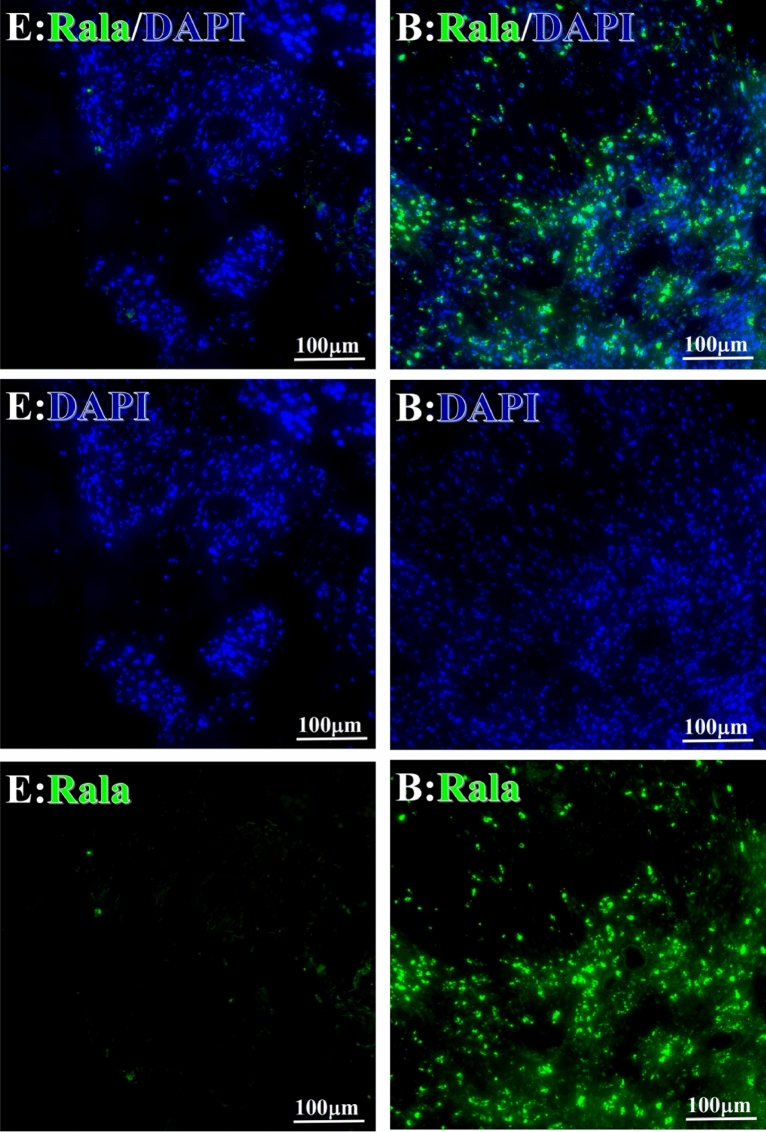
Examples iTAA of Rala fluorescence target binding positive cells before and after primary HELC treatment. Rala: green, DAPI: blue. E: Pathology No. 18054, left lung cancer, biopsy sample from the primary tumor before HELC treatment; B: Pathology No. 18026, esophageal cancer (small cell carcinoma), biopsy sample from abdominal lymph node metastasis which is not treated after primary tumor HELC treatment.

**Figure 9:**
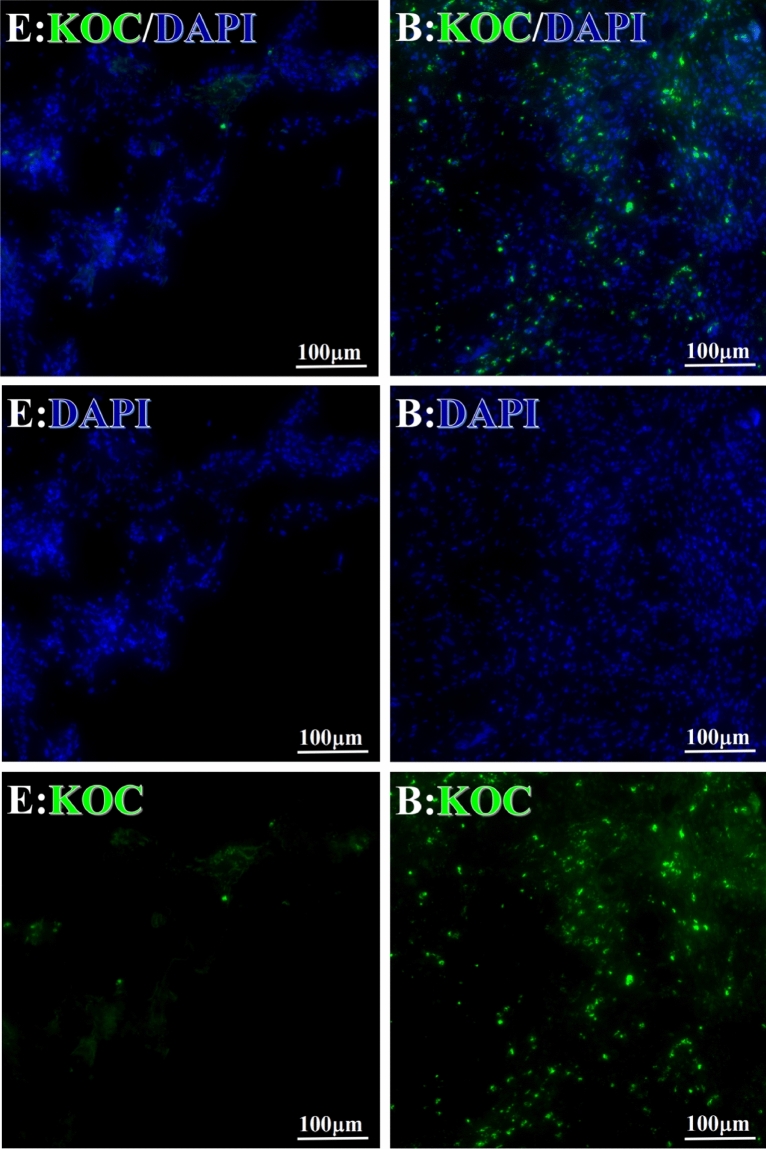
Examples iTAA of KOC fluorescence target binding positive cells before and after primary HELC treatment. KOC: green, DAPI: blue. E: Pathology No. 18054, left lung cancer, biopsy sample from the primary tumor before HELC treatment; B: Pathology No. 18026, esophageal cancer (small cell carcinoma), biopsy sample from abdominal lymph node metastasis which is not treated after primary tumor HELC treatment.

**Figure 10:**
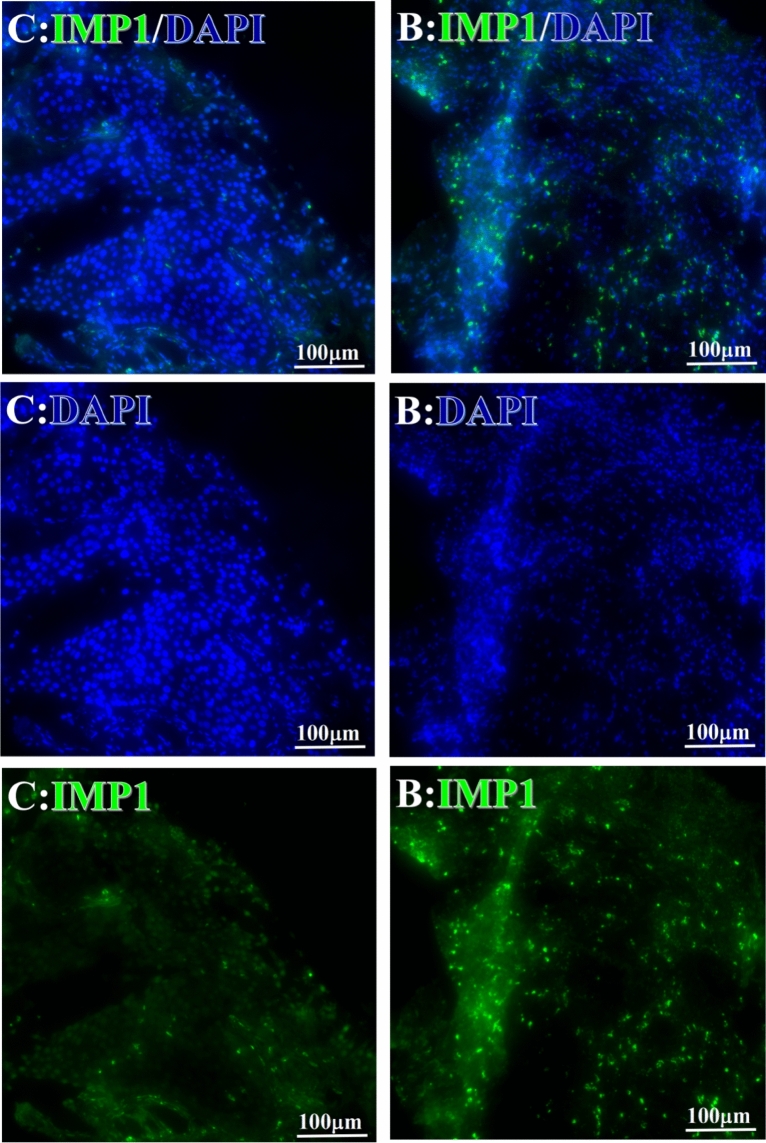
Examples iTAA of INP1 fluorescence target binding positive cells before and after treatment. INP1: Green, DAPI: blue. C: Pathology No. 18050, right lung cancer, biopsy sample from the primary tumor before HELC treatment; B: Pathology No. 18026, esophageal cancer (small cell carcinoma), biopsy sample from abdominal lymph node metastasis which is not treated after primary tumor HELC treatment.

**Figure 11:**
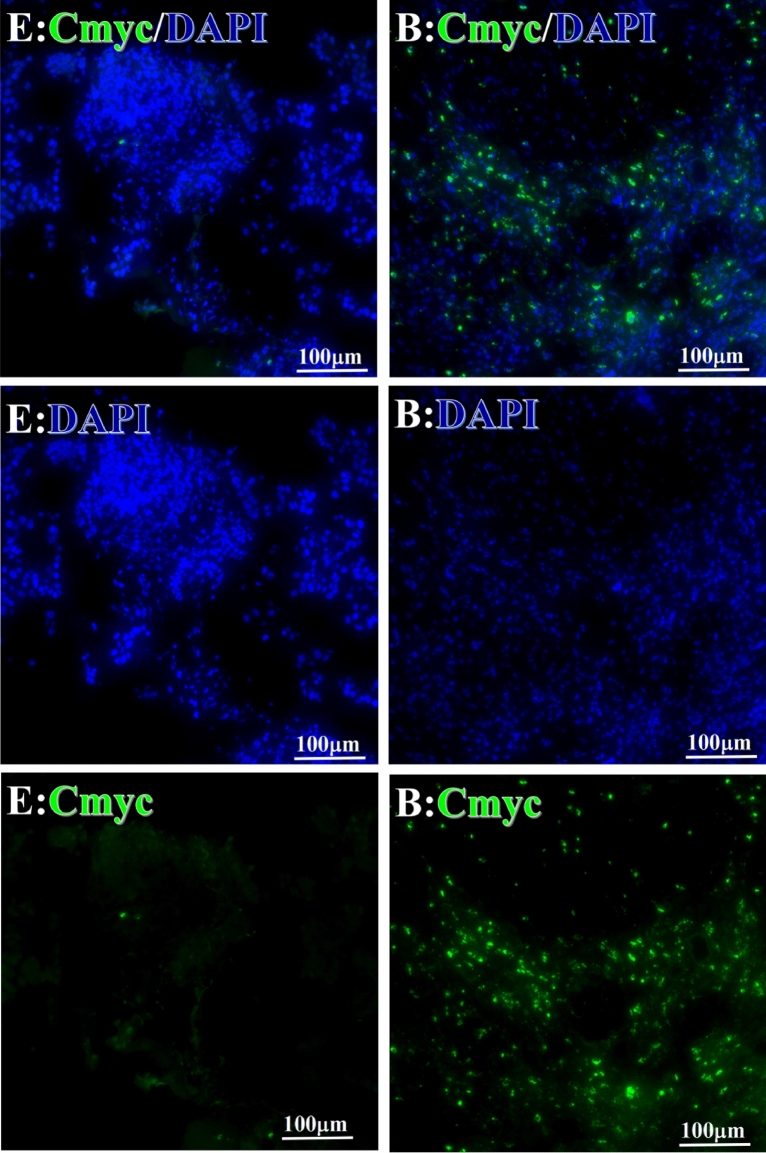
Examples iTAA of Cmyc fluorescence target binding positive cells before and after primary tumor HELC treatment. Cmyc: green, DAPI: blue. E: Pathology No. 18054, left lung cancer, biopsy sample from the primary tumor before HELC treatment; B: Pathology No. 18026, esophageal cancer (small cell carcinoma), biopsy sample from abdominal lymph node metastasis which is not treated after primary tumor HELC treatment.

Before and after treatment, analysis of the positive staining of the complement, the 7 of TAA-bearing fluorescein and mean MFIs were executed for each of iTAAs in all tumor sections to show where each of complement C and iTAAs was being. The percentage of the positive complement was higher after than before for C3 (22.1 ± 6.3 vs. 5.8 ± 3.7, p = 0.03), C5 (57.4 ± 10.2 vs. 4.1 ± 1.5, p < 0.001), and C9 (30.2 ± 9.8 vs. 3.5 ± 1.3, p = 0.005) (Table 3, Figs. 1, 2, 3, 4, 5, 14, 15, 16). Similarly, increased positive rates were seen for the TAAs after versus before comparison for the zeta (36.1 ± 8.1 vs. 4.7 ± 2.0, p = 0.0005), IMP1(25.5 ± 5.2 vs. 2.4 ± 1.8, p = 0.0002), Koc (26.9 ± 7.1 vs. 2.1 ± 1.4, p = 0.0006), Survivin (48.3 ± 5.0 vs. 2.5 ± 1.2, p < 0.0001), C-MYC (38.2 ± 9.8 vs. 3.7 ± 1.4, p = 0.0008), RalA (56.4 ± 9.2 vs. 3.3 ± 1.3, p < 0.0001), and p53 (20.1 ± 7.3 vs. 4.6 ± 2.3, p = 0.03) proteins (Table 3, Figs. 1, 2, 6, 7, 8, 9, 10, 11, 17, 18, 19, 20, 21, 22, 23). The MFI was significantly higher after versus before HELC treatment for the Survivin (21.3 ± 2.8 vs. 2.5 ± 1.0, p < 0.0001), RalA (43.6 ± 6.9 vs. 11.4 ± 4.3, p = 0.0008), and IMP1 (35.3 ± 5.6 vs. 20.1 ± 4.4, p = 0.04) of iTAAs, as well as the complement C, C9 (20.7 ± 5.8 vs. 7.0 ± 1.7, p = 0.02) (Table 3, Figs. 1, 2, 6, 7, 8, 9, 10, 11, 17, 18, 19, 20, 21, 22, 23). This finding illustrates that after patients’ bodies produce the iTAAs, the iTAAs target the TAAs in tumor cells’ nuclei.

**Table 3:** Comparison of target binding rate and MFI of tumor-associated immune autoantibodies positive cells before and after treatment (± SEM).

Name of genes	Ratio of binding	Before treatment	After treatment	*t*	*P*
Zeta	Cell %	4.676 ± 1.985	36.090 ± 8.121	4.416	0.0005
	MFI	17.760 ± 6.678	33.760 ± 8.007	1.536	0.15
Survivn	Cell %	2.514 ± 1.223	48.270 ± 4.977	9.980	< 0.0001
	MFI	2.549 ± 0.984	21.310 ± 2.840	6.814	< 0.0001
Rala	Cell %	3.248 ± 1.436	56.430 ± 9.242	6.805	< 0.0001
	MFI	11.380 ± 4.343	43.600 ± 6.905	4.164	0.0008
KOC	Cell %	2.138 ± 1.363	26.940 ± 7.143	4.235	0.0006
	MFI	7.865 ± 3.846	22.710 ± 7.556	1.936	0.07
IMP1	Cell %	2.421 ± 1.754	25.520 ± 5.159	4.793	0.0002
	MFI	20.060 ± 4.407	35.260 ± 5.527	2.175	0.04
Cmyc	Cell %	3.685 ± 1.401	38.240 ± 9.766	4.199	0.0008
	MFI	3.954 ± 1.904	16.040 ± 3.848	3.085	0.0076
P53	Cell %	4.597 ± 2.251	20.120 ± 7.310	2.347	0.03
	MFI	7.809 ± 6.166	9.363 ± 3.474	0.195	0.85
C3	Cell %	5.768 ± 3.675	22.110 ± 6.329	2.386	0.03
	MFI	19.420 ± 8.299	21.360 ± 5.145	0.179	0.86
C5	Cell %	4.093 ± 1.479	57.380 ± 10.16	6.223	< 0.0001
	MFI	18.870 ± 8.933	30.710 ± 6.066	0.997	0.33
C9	Cell %	3.457 ± 1.326	30.240 ± 9.802	3.251	0.005
	MFI	7.027 ± 1.727	20.740 ± 5.833	2.615	0.02

*N* Nucleus, *C* Perinuclear, Ratio of N to C after treatment, P < 0.05 to 0.001.

### Response and target binding

**Figure 12:**
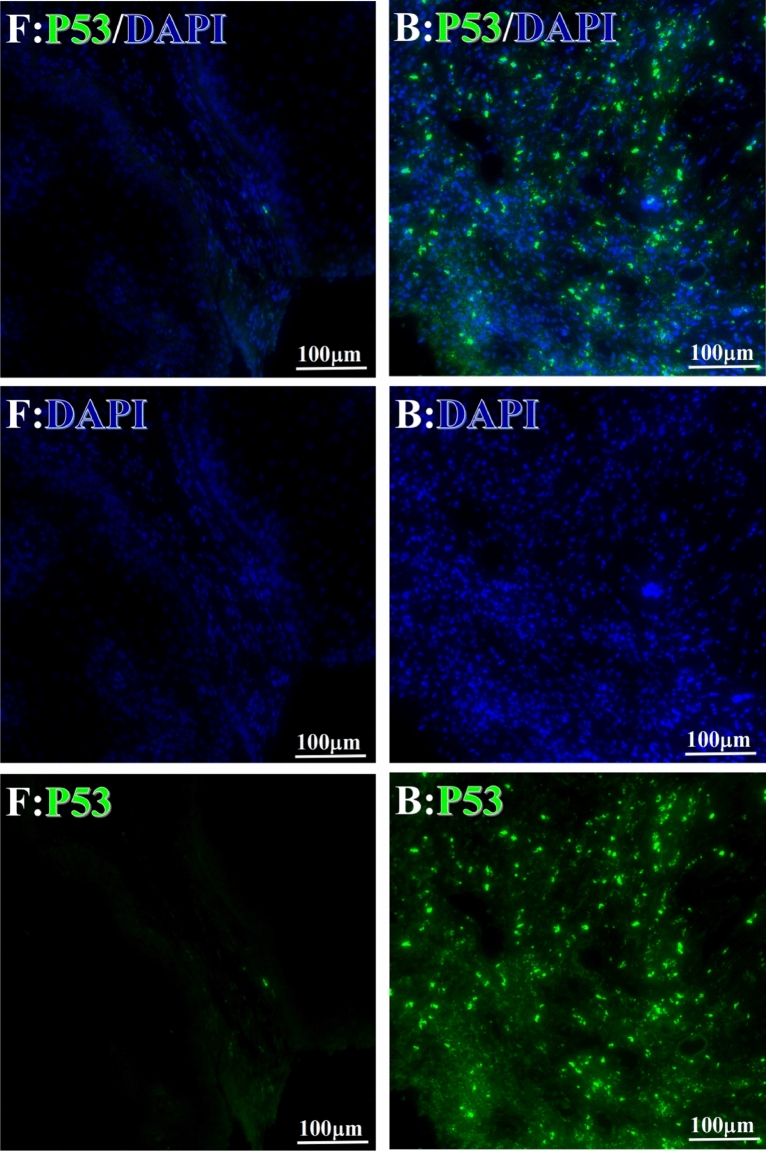
Examples of P53 fluorescence target binding positive cells before and after HELC treatment. P53: Green, DAPI: blue. F: Pathology number 18023, esophageal squamous cell carcinoma, biopsy sample from the primary tumor before HELC treatment. B: Pathology number 18026, esophageal cancer (small cell carcinoma), biopsy sample from lymph node metastasis in the abdominal cavity which is not treated after primary tumor HELC treatment.

**Figure 13:**
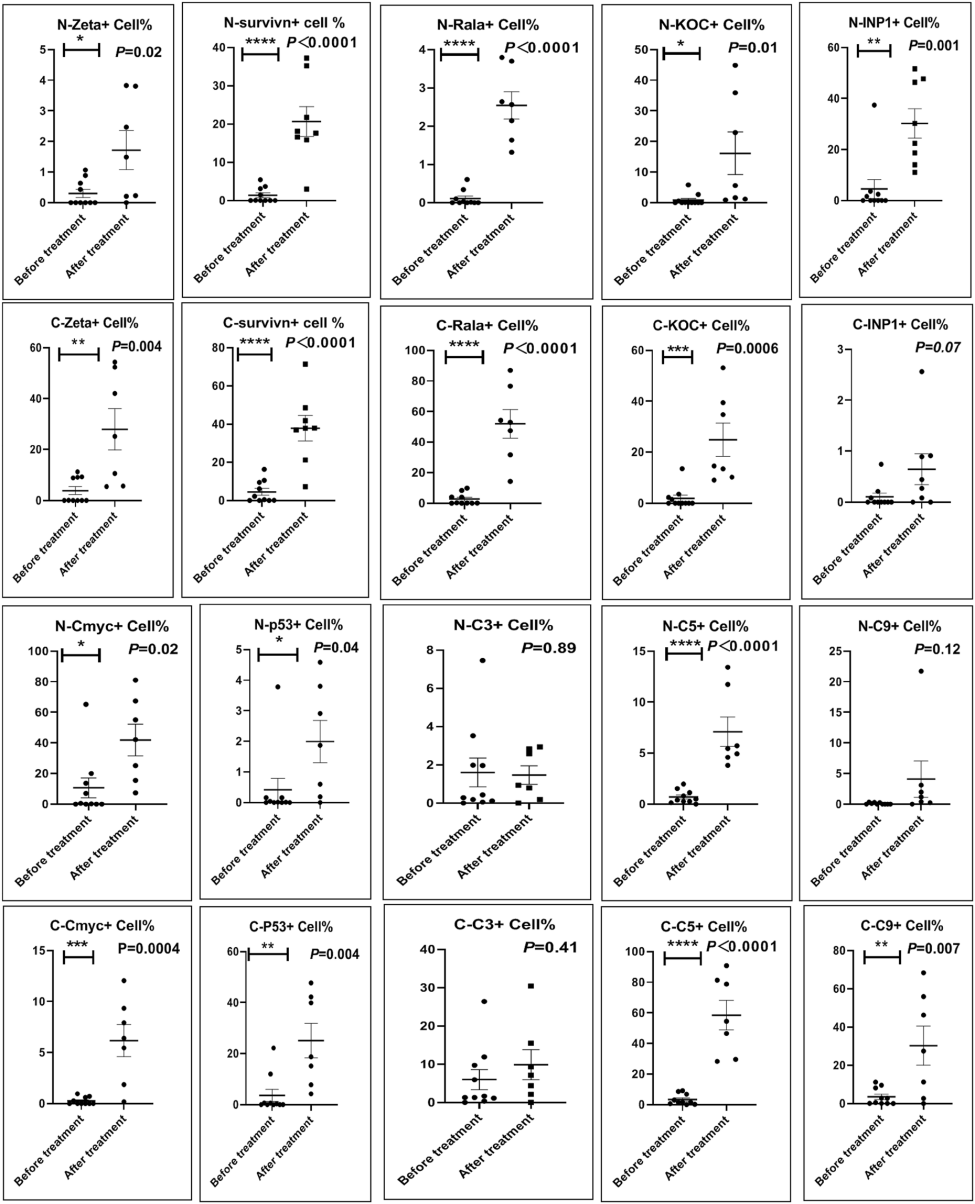
The expression rate of cytoplasmic and nuclear complement positive reaction and the target binding rate of tumor-related immune autoantibodies positive cells before and after treatment. *N* Nuclear, *C* Cytoplasm, *P < 0.05; **P < 0.01, ***P < 0.0001.

Each protein’s intranuclear and partial perinuclear target binding location yielded a corresponding median target binding rate (Table 4, Figs. 6, 7, 8, 9, 10, 17, 18, 19, 20, 21, 22, 23). IMP1 and c-MYC target binding took place mainly in the intranuclear region (26.34%, 95% CI (15.20, 47.39) and 42.2%, 95% CI (15.5, 67.5)) and partially in the perinuclear (0.36%, 95% CI (0.20, 0.91) and 6.41%, 95% CI (1.86, 9.34)) region after treatment (p = 0.001, p = 0.005) (Table 2, Figs. 6, 7, 8, 9, 10, 17, 18, 19, 20, 21, 22, 23). Binding took place mainly in the perinuclear position for the zeta (17.92%, 95% CI (5.52, 49.79)), Survivin (37.95%, 95% CI (25.13, 4.90)), RalA (52.94%, 95% CI (39.57, 65.46)), Koc (14.57%, 95% CI (11.86, 37.16)), and p53 (18.76%, 95% CI (7.76, 42.28)) proteins (p = 0.0008, p = 0.04, p = 0.0002, p = 0.38, p = 0.005) (Table 4, Figs. 6, 7, 8, 9, 10, 17, 18, 19, 20, 21, 22, 23). The target binding rate for zeta at the intranuclear (1.98%, 95% CI (0.22, 3.83)) and perinuclear (17.92%, 95% CI (5.52, 49.79)) site was low (p = 0.04, p = 0.0008). Conversely, Survivin had a higher target binding rate in the nucleus (17.89%, 95% CI (16.07, 31.86)) and cytoplasm (37.95%, 95% CI (25.13, 4.90)) than nearly all the protein factors measured (p = 0.12, p = 0.04) (Table 4, Figs. 6, 7, 8, 9, 10, 17, 18, 19, 20, 21, 22, 23). The RalA target binding rate in the nucleus (2.57%, 95% CI (2.57, 3.17)) was low; however, its perinuclear (52.94, 95% CI (39.57, 65.46)) site’s binding rate was the most significant of all the proteins tested (p = 0.04, p = 0.0002) (Table 4, Figs. 6, 7, 8, 9, 10, 17, 18, 19, 20, 21, 22, 23). The Koc target binding rate in the nucleus (5.61%, 95% CI (1.41, 29.45)) and the cytoplasm (14.57%, 95% CI (11.86, 37.16)) was relatively low (p = 0.44, p = 0.38) (Table 2, Figs. 6, 7, 8, 9, 10, 17, 18, 19, 20, 21, 22, 23). The p53 iTAA target binding rates showed a similar trend in both the intranuclear (1.87%, 95% CI (0.19, 3.80)) and the perinuclear (18.76%, 95% CI (7.76, 42.28)) location (p = 0.21, p = 0.005) (Table 4, Figs. 6, 7, 8, 9, 10, 12, 13, 17, 18, 19, 20, 21, 22, 23, 24).

**Table 4:** Comparison of intranuclear and perinuclear positive staining and targeting ratios before and after each factor treatment.

	Time	N- cell %	C- cell %	*P*
Zeta	Before treatment	0.00 (0.00, 0.70)	0.04 (0.00, 9.17)	0.04
	After treatment	1.98 (0.22, 3.83)	17.92 (5.52, 49.79)	0.008
*P*		0.02	0.004	
Survivn	Before treatment	0.04 (0.00, 3.23)	1.32 (0.00, 9.69)	0.12
	After treatment	17.89 (16.07, 31.86)	37.95 (25.13, 4.90)	0.04
*P*		< 0.0001	< 0.0001	
Rala	Before treatment	0.005 (0.00, 0.08)	0.23 (0.06,3.97)	0.04
	After treatment	2.57 (1.90, 3.17)	52.94 (39.57, 65.46)	0.0002
*P*		< 0.0001	< 0.0001	
KOC	Before treatment	0.02 (0.00, 0.32)	0.01 (0.00, 1.88)	0.44
	After treatment	5.61 (1.41, 29.45)	14.57 (11.86, 37.16)	0.38
*P*		0.01	0.0006	
IMP1	Before treatment	0.01 (0.00, 2.76)	0.00 (0.00, 0.11)	0.25
	After treatment	26.34 (15.20, 47.39)	0.36 (0.20, 0.91)	0.0001
*P*		0.001	0.07	
Cmyc	Before treatment	0.35 (0.033, 15.26)	0.00 (0.00, 0.63)	0.12
	After treatment	42.21 (15.53, 67.46)	6.41 (1.86, 9.34)	0.005
*P*		0.02	0.0004	
P53	Before treatment	0.01 (0.00, 0.16)	0.18 (0.00, 3.49)	0.21
	After treatment	1.87 (0.19, 3.80)	18.76 (7.76, 42.28)	0.005
*P*		0.04	0.003	
C3	Before treatment	0.36 (0.10, 2.37)	1.48 (0.93, 10.26)	0.13
	After treatment	0.93 (0.19, 2.83)	7.16 (2.12, 15.50)	0.06
*P*		0.89	0.41	
C5	Before treatment	0.46 (0.24, 1.23)	1.53 (0.51, 7.23)	0.04
	After treatment	5.45 (4.60, 1.74)	54.46 (29.66, 81.41)	0.0002
*P*		< 0.0001	< 0.0001	
C9	Before treatment	0.005 (0.00, 0.22)	1.59 (0.09, 6.81)	0.02
	After treatment	1.17 (0.33, 2.54)	27.45 (6.93, 51.14)	0.03
*P*		0.12	0.007

**Figure 14:**
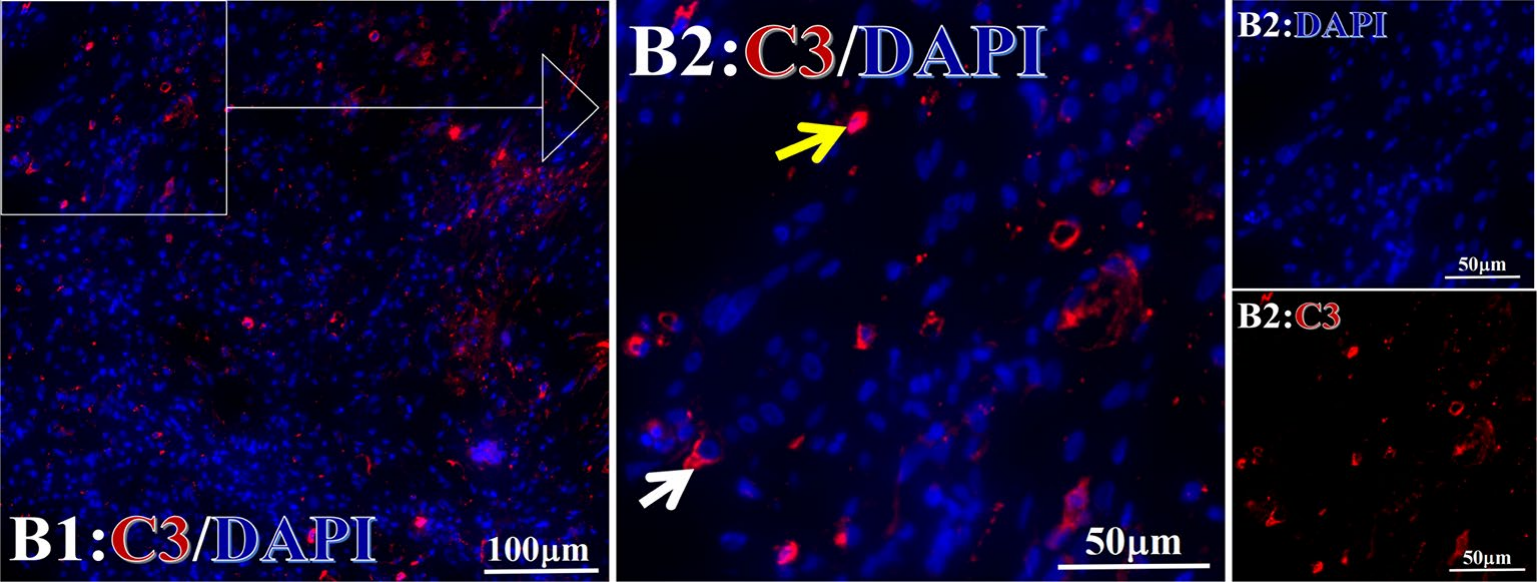
Example of the reaction position of complement C3 positive reaction. C3: red, DAPI: blue. B: Pathology number 18026, esophageal cancer (small cell carcinoma), biopsy sample from lymph node metastasis in the abdominal cavity which is not treated after primary tumor HELC treatment. B1: Bar = 100 µm; B2: magnified image of B1, Bar = 50 µm. White arrows are examples of cytoplasmic positive reaction expression, and yellow arrows are examples of nuclear positive reaction.

**Figure 15:**
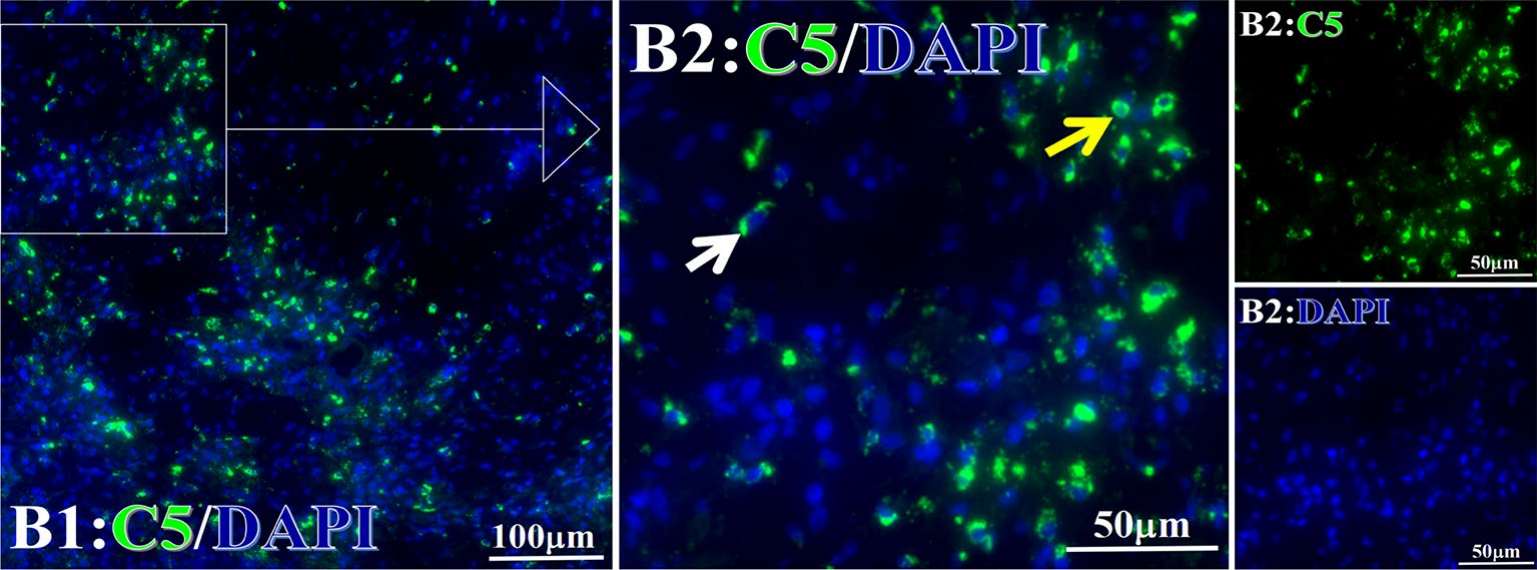
Example of the reaction position of complement C5 positive reaction. C5: Green, DAPI: blue. B: Pathology number 18026, esophageal cancer (small cell carcinoma), biopsy sample from lymph node metastasis in the abdominal cavity which is not treated after primary tumor HELC treatment. B1: Bar = 100 µm; B2: magnified image of B1, Bar = 50 µm. White arrows are examples of cytoplasmic positive reaction, and yellow arrows are examples of nuclear positive reaction.

**Figure 16:**
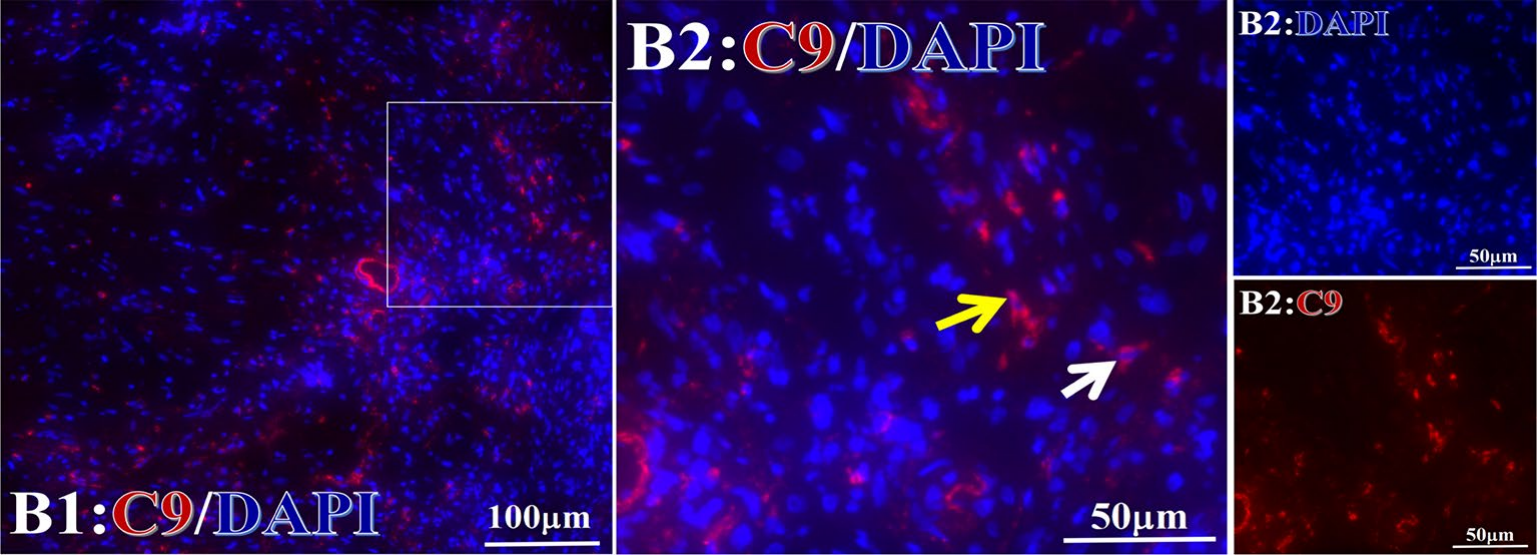
Example of the reaction position of complement C9 positive reaction. C9: red, DAPI: blue. B: Pathology number 18026, esophageal cancer (small cell carcinoma), biopsy sample from lymph node metastasis in the abdominal cavity which is not treated after primary tumor HELC treatment. B1: Bar = 100 µm; B2: magnified image of B1, Bar = 50 µm. White arrows are examples of cytoplasmic positive reaction, and yellow arrows are examples of nuclear positive reaction.

Complement C3, C5 and C9 were found primarily in the perinuclear region. C3’s reaction position, partially in the nucleus, was stained both in the intranuclear (0.93%, 95% CI (0.19, 2.83)) and perinuclear (7.16%, 95% CI (2.12, 15.50)) regions (p = 0.06) (Table 4, Figs. 3, 4, 5, 6, 14, 15, 16). The median target binding rates of C5 were much higher than C3 in the intranuclear (5.45%, 95% CI (4.60, 1.74)) and perinuclear (54.46%, 95% CI (29.66, 81.41)) sites (p = 0.0002). C9’s median target bound intranuclear (1.17%, 95% CI (0.33, 2.54)) and perinuclear (27.45%, 95% CI (6.93, 51.14)) site values rest between the complements tested (p = 0.03) (Table 4, Figs. 3, 4, 5, 6, 14, 15, 16).

**Figure 17:**
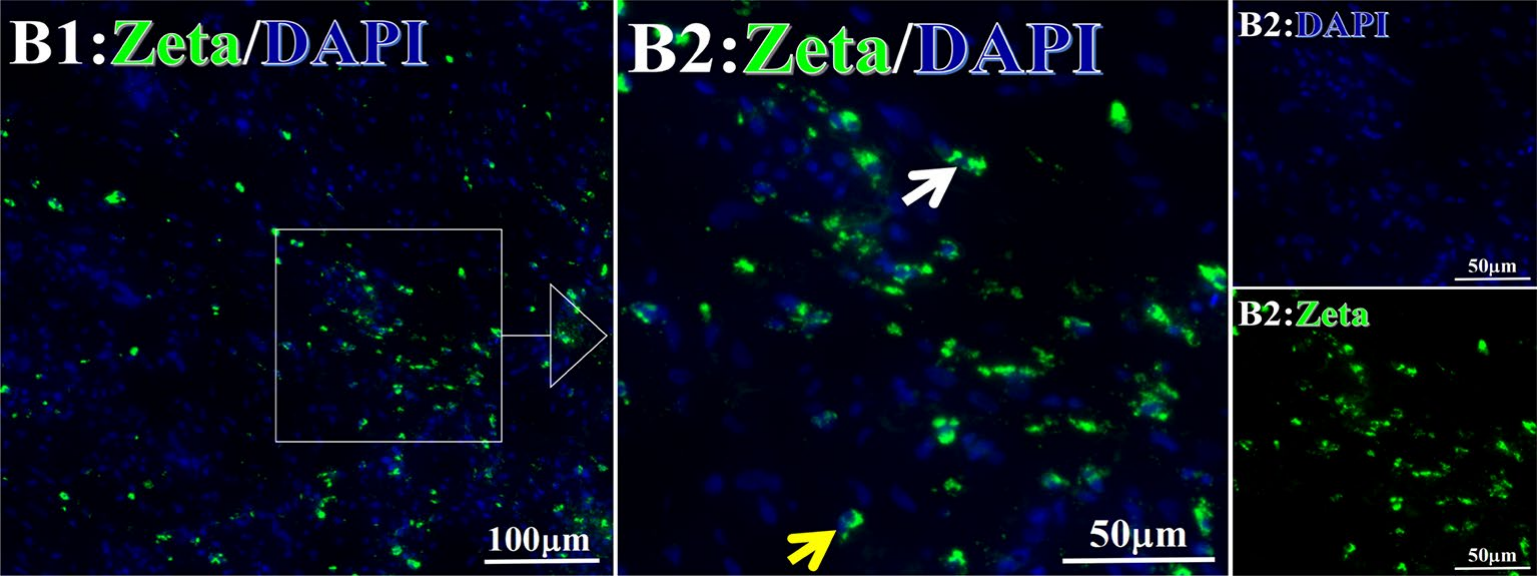
Example map of target binding position for iTAA of Zeta positive cells. Zeta: Green, DAPI: blue. B: Pathology number 18026, esophageal cancer (small cell carcinoma), biopsy sample from lymph node metastasis in the abdominal cavity which is not treated after primary tumor HELC treatment. B1: Bar = 100 µm; B2: magnified image of B1, Bar = 50 µm. White arrows are examples of cytoplasmic target binding positive, and yellow arrows are examples of target binding nuclear positive.

**Figure 18:**
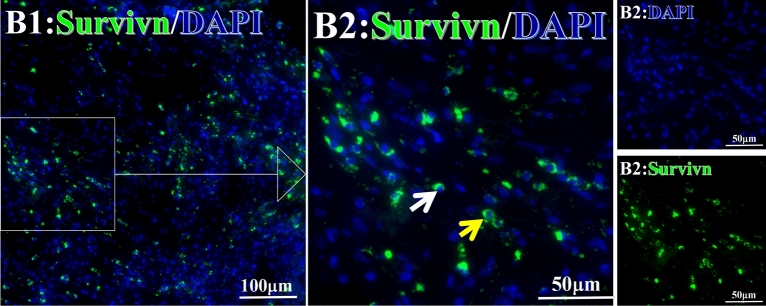
Example map of target binding position for iTAA of Survivn positive cells. Survivn: Green, DAPI: blue. B: Pathology number 18026, esophageal cancer (small cell carcinoma), biopsy sample from lymph node metastasis in the abdominal cavity which is not treated after primary tumor HELC treatment. B1: Bar = 100 µm; B2: magnified image of B1, Bar = 50 µm. White arrows are examples of cytoplasmic target binding positive, and yellow arrows are examples of target binding nuclear positive.

**Figure 19:**
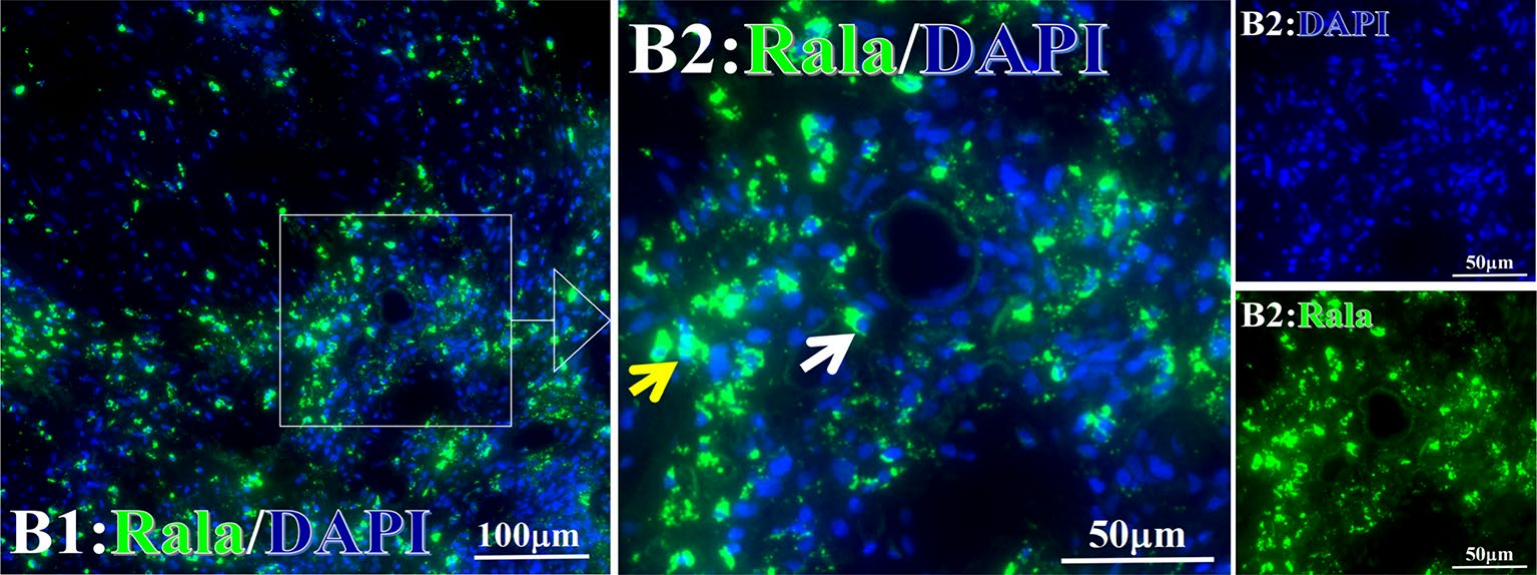
Example map of targeted binding position for iTAA of Rala positive cells. Rala: Green, DAPI: blue. B: Pathology number 18026, esophageal cancer (small cell carcinoma), biopsy sample from lymph node metastasis in the abdominal cavity which is not treated after primary tumor HELC treatment. B1: Bar = 100 µm; B2: magnified image of B1, Bar = 50 µm. White arrows are examples of cytoplasmic target binding positive, and yellow arrows are examples of target binding nuclear positive.

**Figure 20:**
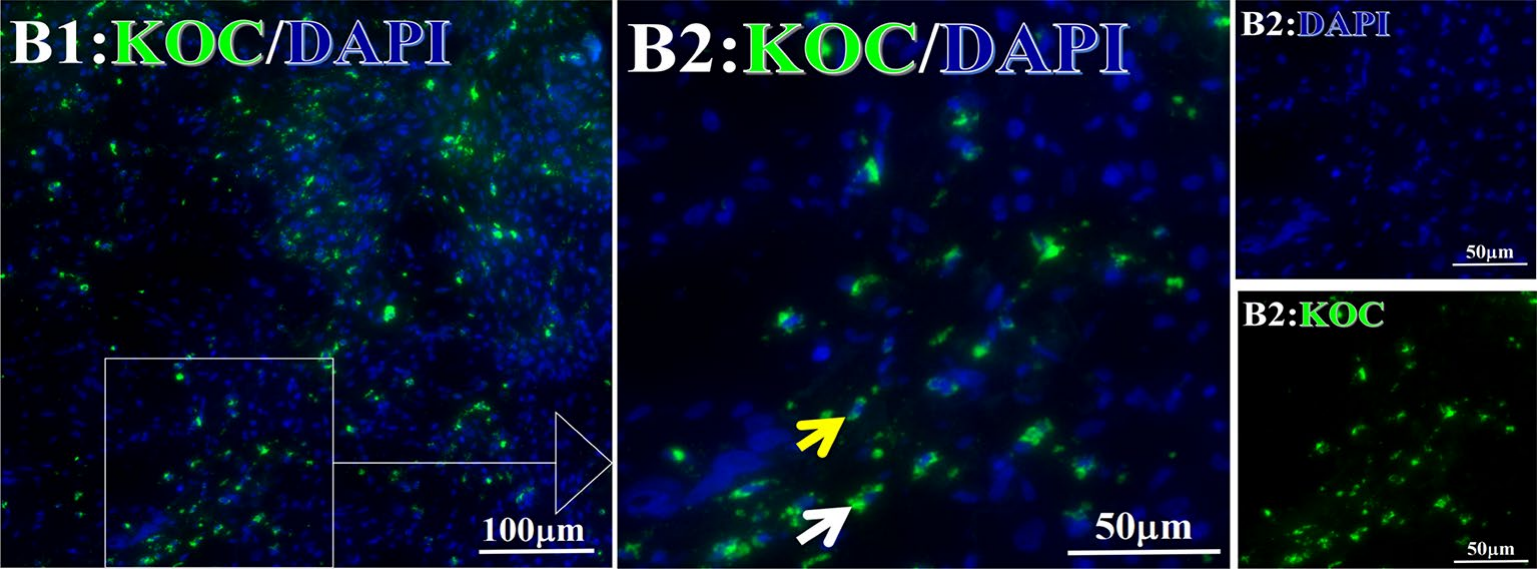
Example map of target binding position for iTAA of KOC-positive cells. KOC: Green, DAPI: blue. B: Pathology number 18026, esophageal cancer (small cell carcinoma), biopsy sample from lymph node metastasis in the abdominal cavity which is not treated after primary tumor HELC treatment. B1: Bar = 100 µm; B2: magnified image of B1, Bar = 50 µm. White arrows are examples of cytoplasmic target binding positive, and yellow arrows are examples of nuclear target binding positive.

**Figure 21:**
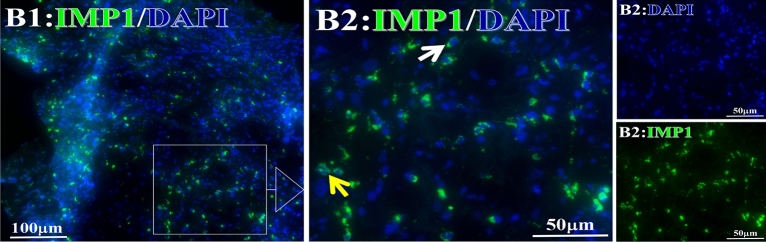
Example map of target binding position for iTAA of INP1-positive cells. INP1: Green, DAPI: blue. B: Pathology number 18026, esophageal cancer (small cell carcinoma), biopsy sample from lymph node metastasis in the abdominal cavity which is not treated after primary tumor HELC treatment. B1: Bar = 100 µm; B2: magnified image of B1, Bar = 50 µm. White arrows are examples of cytoplasmic target binding positive, and yellow arrows are examples of nuclear target binding positive.

**Figure 22:**
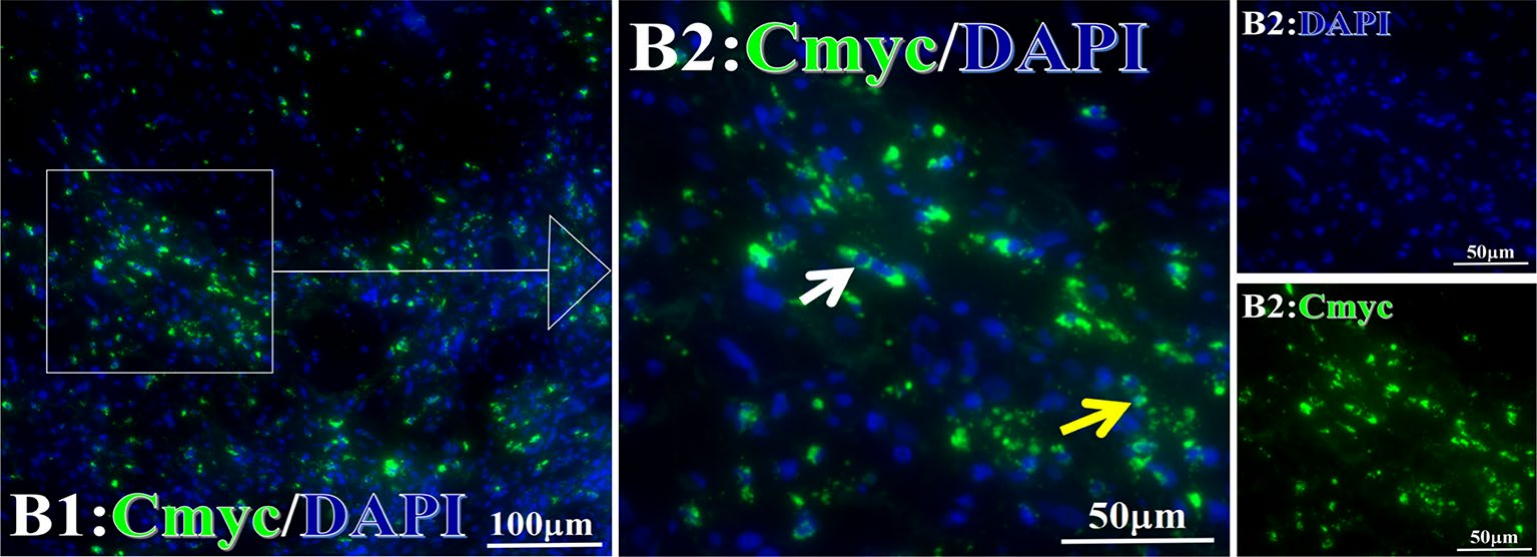
Example map of target binding position for iTAA of Cmyc positive cells. Cmyc: Green, DAPI: blue. B: Pathology number 18026, esophageal cancer (small cell carcinoma), biopsy sample from lymph node metastasis in the abdominal cavity which is not treated after primary tumor HELC treatment. B1: Bar = 100 µm; B2: magnified image of B1, Bar = 50 µm. White arrows are examples of cytoplasmic target binding positive, and yellow arrows are examples of nuclear target binding positive.

**Figure 23:**
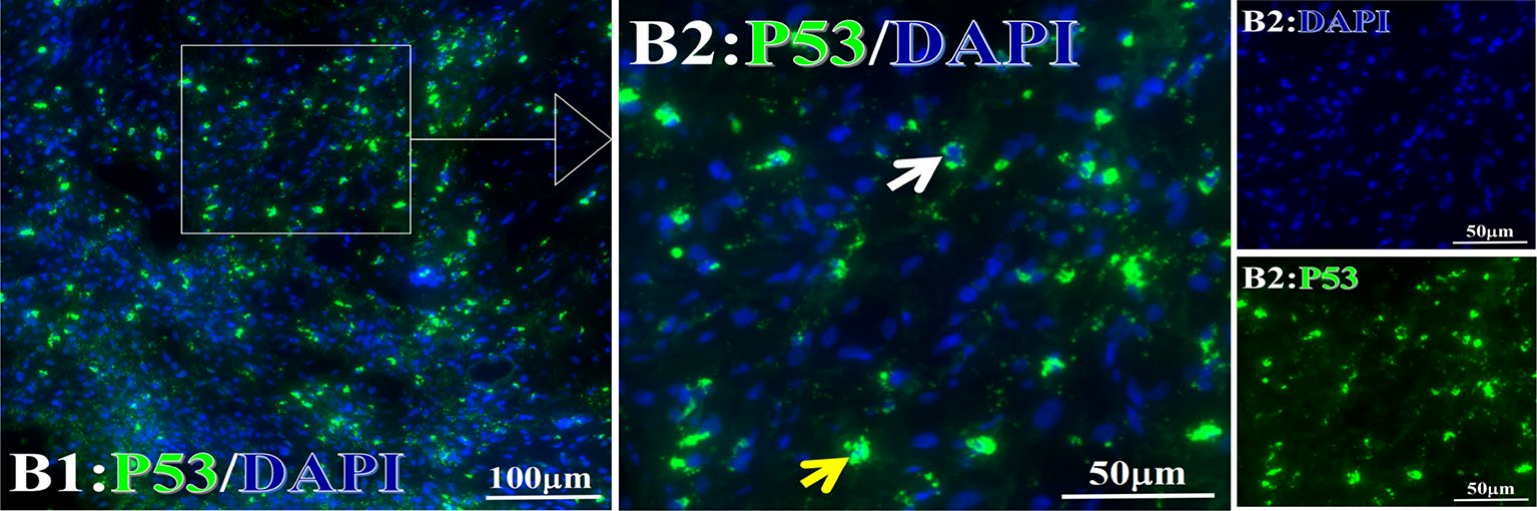
Example map of target binding position for iTAA of P53 positive cells. P53: Green, DAPI: blue. B: Pathology number 18026, esophageal cancer (small cell carcinoma), biopsy sample from lymph node metastasis in the abdominal cavity which is not treated after primary tumor HELC treatment. B1: Bar = 100 µm; B2: magnified image of B1, Bar = 50 µm. White arrows are examples of cytoplasmic target binding positive, and yellow arrows are examples of nuclear target binding positive.

In the analysis of the iTAAs’ targeting binding locations and levels before and after HELC chemotherapy in the intranuclear regions of the tumor cells, the c-MYC (0.35%, 95% CI (0.033, 15.26) vs. 42.21%, 95% CI (15.53, 67.46)), IMP1 (0.01%, 95% CI (0.00, 2.76) vs. 26.34%, 95% CI (15.20, 47.39)) and Survivin (0.04%, 95% CI (0.00, 3.23) vs. 17.89%, 95% CI (16.07, 31.86)) proteins were significantly elevated (p = 0.02, p = 0.001, p < 0.0001) (Table 4, Figs. 6, 7, 8, 9, 10, 17, 18, 19, 20, 21, 22, 23). In contrast, a substantial increase in the perinuclear region was seen for zeta (0.00%, 95% CI (0.00, 0.70) vs. 1.98% CI (0.22, 3.83)), Koc (0.02%, 95% CI (0.00, 0.32) vs. 5.61% CI (1.41, 29.45)), RalA (0.005%, 95% CI (0.00, 0.08) vs. 2.57% CI (1.90, 3.17)), and p53 (0.01%, 95% CI (0.00, 0.16) vs. 1.87% CI (0.19, 3.80)) (p = 0.02, p = 0.01, p < 0.0001, p = 0.04) (Table 4, Figs. 6, 7, 8, 9, 10, 17, 18, 19, 20, 21, 22, 23). The target binding rates reveal that different iTAAs can bind in a different original cellular position in tumor cells, either perinuclear or intranuclear. This binding process may take part in the regulation of tumor cell growth in a different way than it does for control of tumor growth.

## Discussion

**Figure 24:**
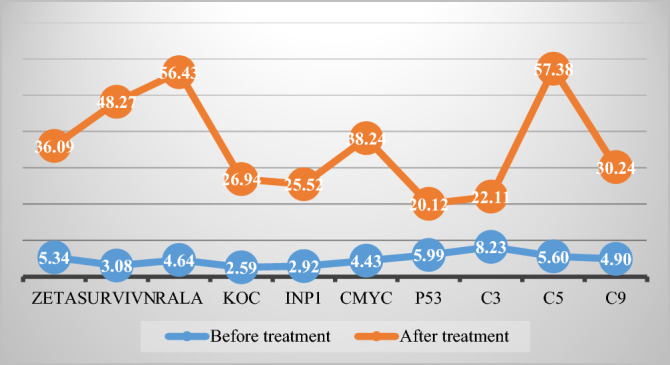
Trend analysis of expression autoantibodies aTAA before treatment and iTAA after treatment, complement levels before and after treatment.

The aTAAs are often produced in the human body but maintain a low-level presence^8,11^. The number of aTAAs may surge if a virus mutates the TAAs^11^. The mutation can occur when viruses or mutated proteins from oncogenes or genes other than those originating from the human body cause tumors^8,11^. Otherwise, aTAAs can be generated through the injection of hapten modified with TAA. This modified TAA possesses a slight change on its epitope so it is recognized by the immune system as a neoantigen, and thus incites a humoral response^4,11,20^. Moreover, an antibody’s amplification response to the presence of an antigen means that even a small quantity of antigens in the early stage of tumorigenesis can trigger a relatively large immune response^4,22^. Therefore, aTAAs are feasible early diagnostic markers. Despite using aTAAs as biomarkers for clinical diagnosis, the iTAAs should be studied in detail for application to cancer treatments (Fig. 24).

**Figure 25:**
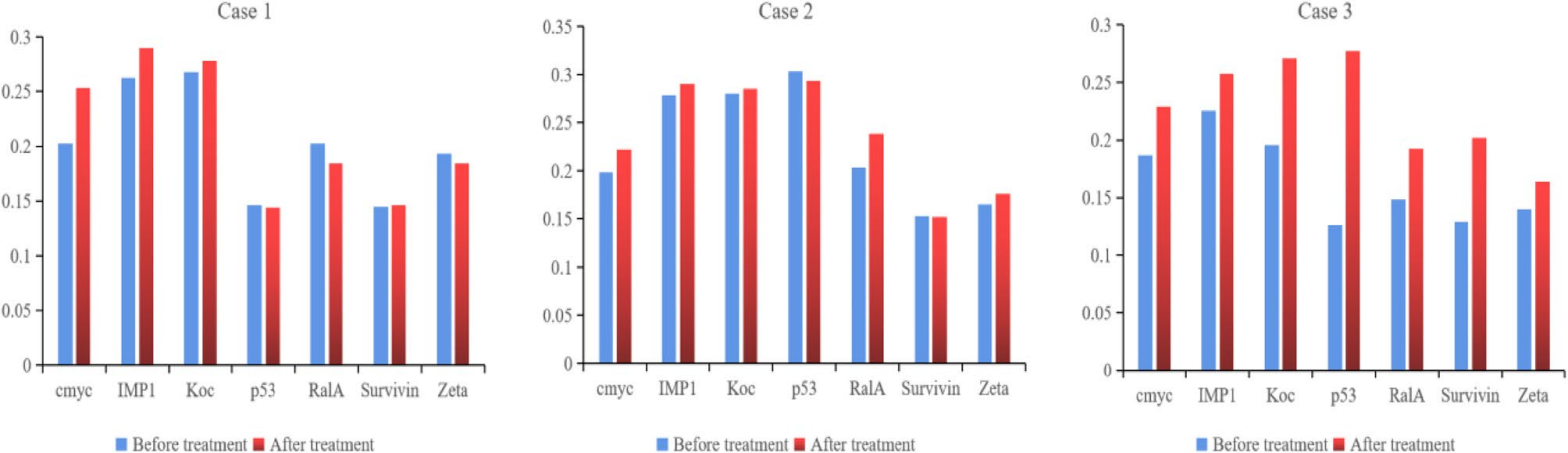
The level of aTAA before treatment and iTAA after treatment.

We established that HELC therapy induces an immune response from tumors with DC, CD4 and CD8 positive tumor tissue after treatment^4,9,10^. We found a dendritic cell (DC) using an electric microscope and the DC11b/c expression was increased in our tumor mice model^4,9,10^. DC and T cells of whole immunity systems were activated following HELC. Therefore, we believe that B cells of immunity systems must be activated as well at same times, so the iTAAs are produced following immunity reaction induced by neu TAA in the patient. Follow the iTAAs’ activated production, they analyze, target, and then bind to tumor cells. Thus, this study confirms that the iTAAs are induced and excited in the sera after HELC treatment (Table 2, Fig. 25), and iTAAs can circulate from blood to bind the tumor at the cellular level (Figs. 6, 7, 8, 9, 10, 11) since the iTAA has a high specificity to tumor cells, while the aTAAs can not bind tumor cells. This study confirms that the complement C3, C5 and C9 were primarily staining the perinuclear region (Table 4, Fig. 5). The C5 and C9 reactions on the surface of tumor cells to punch hole and associate for iTAA to enter the intracellular and intranuclear region of tumor cells. This study is the first to use IF TAA-bearing fluorescence to detect the iTAAs in tumor cells rather than diagnostic detect aTAAs as biomarkers in circulation^20^.

Since the aTAAs were not found an increase and high specificity binding of in the patients’ tumor cells before treatment of HELC, further confirmation emerges supporting the assertion that the iTAAs increase in presence with a higher specificity after the treatment courses and target critical locations. The IMP1, c-MYC, and Survivin increased significantly in the intranuclear tumor cell locations. In contrast, Zeta, Koc, RalA, and p53 were increased considerably in the perinuclear sites, and the target binding rate revealed that different iTAAs could bind to different original cellular positions in tumor cells after treatment, which could play a unique role in the regulation of tumor cell growth (Table 4). The seven iTAAs we studied are representative of countless tumor gene products induced by hapten (Tables 3, 4). More gene products may be able to induce more significant quantities the iTAAs. Those representatives of zeta, IMP1, Koc, Survivin, c-MYC, RalA, and p53 gene were studied for iTAAs changes, it is not enough to show the full picture due to different gene with different function, for example zeta is an regulatory effect on angiogenesis and cancer; IMP1 promotes tumor growth; KOC is a novel onco-foetal gene indicator of malignancy; Survivin is essential for cell division and can inhibit cell death; c-myc is of great importance in controlling cell growth and vitality; RALA is highly homologous small G proteins belonging to the RAS superfamily; p53 is the most frequently mutated gene across all cancer types, its functions has evolved since its discovery four decades ago, current knowledge of p53 functions derived through the major classes of anti-p53 antibodies, which could be a paradigm for understanding other molecular events in health and disease^23–29^. However, the method used is an indirect of assess the signals of iTAAs for 7 maker genes as representatives, so that limitations of our study approach needs to improve in the future study.

A hypothesis is that iTAAs bind the TAAs in nuclear of tumor cells may feedback to regulate the expression of different genes depending on what genes function while the iTAAs bind the TAA in blood could do nothing. The iTAAs, the study’s results establish that, once induced, iTAAs circulate in the blood to search for the primary tumor or metastasis site to bind to the nuclei of those tumor cells. We can propose that this process is a result of an abscopal effect.

Investigation of how these iTAA-bound tumor cells survive, die faster, live better, or live differently is required. Future studies might involve collecting circulating tumor cells (CTCs) from cancer patients’ post-HELC treatment and then culturing the CTCs for analysis with IF using TAA-bearing fluorescein, followed by analysis of how the CTCs live. Through sequencing and proteomics, evaluation of the expression of TAAs’ different proteins, including their DNA and RNA, is critical. Finally, further detailed studies, with and without hapten-enhanced intratumoral injections in patients are required to develop a comprehensive understanding of the full diagnostic and therapeutic potential of the iTAAs.

## Data Availability

The data that support the findings of this study are available from [third party name] but restrictions apply to the availability of these data, which were used under license for the current study, and so are not publicly available. Data are however available from the authors upon reasonable request and with writing permission of [third party name] to Baofa Yu.
